# Derkash's Classification and Vas Visual Analog Scale to Access the Long-Term Outcome of Neurothoracic Outlet Syndrome: A Meta-Analysis and Systematic Review

**DOI:** 10.3389/fneur.2022.899120

**Published:** 2022-07-06

**Authors:** Wei Lingyun, Sha Ke, Zhao Jinmin, Qiao Yu, Qin Jun

**Affiliations:** ^1^Research Centre for Regenerative Medicine, Orthopaedic Department, The First Affiliated Hospital of Guangxi Medical University, Nanning, China; ^2^Guangxi Key Laboratory of Regenerative Medicine, Guangxi Medical University, Nanning, China; ^3^Collaborative Innovation Centre of Regenerative Medicine and Medical Bio Resource Development and Application Co-constructed by the Province and Ministry, Nanning, China; ^4^Department of Traumatic Hand Surgery, The First Affiliated Hospital of Guilin Medical College, Guilin, China

**Keywords:** neurologic thoracic outlet syndrome, vas visual analog scale, long-term outcome, meta-analysis, surgical approach (supraclavicular or trams auxiliary)

## Abstract

**Objective:**

Many publications report outcomes of surgical treatment for neurothoracic outlet syndrome (NTOS); however, high-quality meta-analyses regarding objective evaluation system accessing the long-term outcome of NTOS are lacking. This meta-analysis summarizes and compares the outcomes of Derkosh's classification and vas visual analog scale of the supraclavicular neuroplastic of brachial plexus (SNBP) and trams auxiliary first rib resection (TFRR).

**Methods:**

The Cochrane Library, PubMed, EMBASE, Allied and Complementary Medicine (AMED) were searched for papers published between January 1980 and February 2021, using the keywords “thoracic outlet syndrome,” “treatment, surgical.” Articles were eligible for inclusion if the following criteria were met studies describing outcomes of surgery for NTOS, published in English, human studies, and available in full text. The exclusion criteria were case reports (*n* < 10), reviews, abstracts, and studies lacking a control group or without evaluation for two types of surgery.

**Results:**

We included 10 studies with 1,255 cases, out of which 622 were in the SNBP group; and 633 were in the TFRR group. After surgery (≥12 months), Derkash's classification was improved in 425 cases with SNBP and 364 cases with TFRR. OR = 1.34 (95% CI: 0.94, 1.92), *P* = 0.03; vas visual analog scale was improved in 282 cases in the SNBP group and 214 cases in the TFRR group. OR = 1.08 (95% CI: 0.63, 1.85), *P* = 0.78.

**Conclusion:**

This meta-analysis shows that both SNBP and TFRR are effective for NTOS, but that SNBP is better than TFRR in improving Derkash's classification in the long term. Although patients treated with SNBP are more satisfactory, there is no significant difference in vas visual analog scale from TFRR.

**Systematic Review Registration:**

https://www.crd.york.ac.uk/PROSPERO/display_record.asp?ID=CRD42021254203, PROSPERO CRD42021254203.

## Introduction

Neurological thoracic outlet syndrome (NTOS) accountsfor ~80%−97% of patients with thoracic outlet syndrome (TOS) (1, 2). Treating priorities with rehabilitation training and analgesia can significantly improve outcomes for most patients (3–5). Surgical treatment may be considered for patients whose conservative treatment was not effective or worsened for more than 6 months (6). Most clinicians believe that surgery has a positive effect on the rapid recovery of the upper limb, pain relief, and low recurrence (7). The surgical treatments mainly include the supraclavicular neuroplastic of brachial plexus (SNBP) and trams auxiliary first rib resection (TFRR). Yin et al. (8) conducted a meta-analysis on the outcome of SNBP and TFRR through 32 studies. The conclusion showed that the SNBP has a high probability of success rate >80% and the TFRR has a high probabilities of success rate >70% but only low probabilities of success rate >80%. However, this review was complicated by lacking quantifiable measures of symptoms and disabilities using validated patient-reported instruments. Previous studies have neglected the access criterion about long-term outcomes. Thus, these results are inadequate for evaluation.

We conducted a meta-analysis with the primary objective of comparing Derkash's classification to access the long-term outcome of NTOS after surgery. Our secondary objective was to compare the vas visual analog scale after surgery and to analyze the reasons for the difference between the two.

## Material And Methods

### Strategy

This meta-analysis was based on Preferred Reporting Items for Systematic Reviews and Meta-Analyses (PRISMA) guidelines (9). This study has been registered with PROSPERO (https://www.crd.york.ac.uk/PROSPERO/display_record.asp?ID=CRD42021254203).

### Literature Search

Two reviewers (W.L.Y and Q.Y) independently conducted a literature search and cross-check. Discussion took place with the third investigator (S.K and Z.J.M) when they had different opinions. We searched the Cochrane Library, PubMed, EMBASE, Allied and Complementary Medicine (AMED) through the network. To avoid missing valid documents, we further searched conference reports and unpublished documents. The keywords: (thoracic outlet syndrome) and (treatment, surgical) were searched from January 1980 to February 2021. Free text words were also used instead of MeSH terms to avoid missing recent publications that were not yet given MeSH headings.

### Study Selection

**Figure 1:**
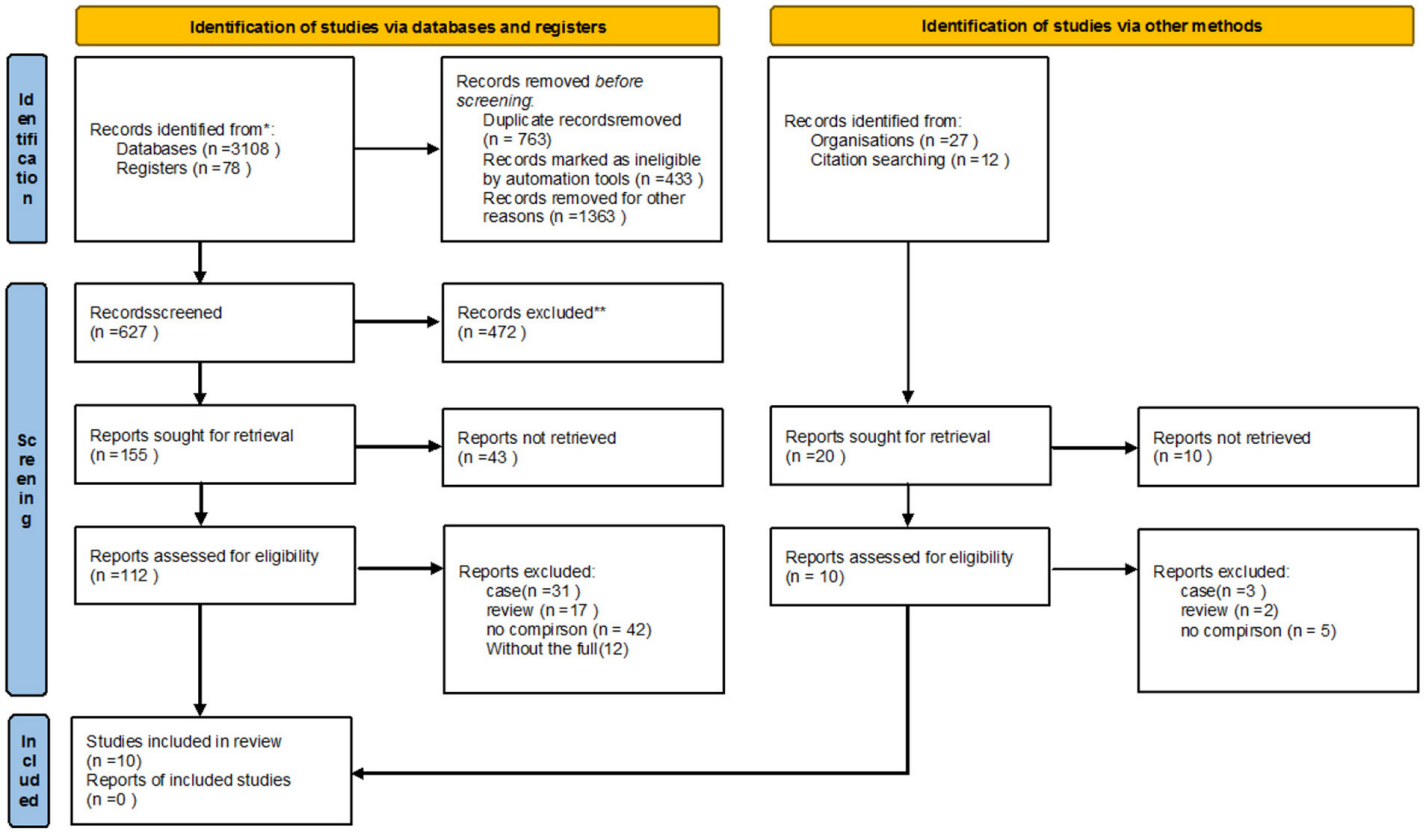
The study identification process. *Consider, if feasible to do so, reporting the number of records identified from each database or register searched (rather than the total number across all databases/registers). **If automation tools were used, indicate how many records were excluded by a human and how many were excluded by automation tools.

The review identified 3,225 articles. After removal of duplicates and ineligible articales, 647 remained (Figure 1). Two authors (W.L.Y and Q.Y) screened the titles and abstracts of the identified studies for relevance.Full texts were obtained of the remaining relevant studies, and two authors (W.L.Y and S.K) read the full-text papers for 175 studies, and a third reviewer (Z.J.M) resolved any disagreements with a final selection. Ten articles that met the inclusion criteria for qualitative analysis were retained.

### Data Extraction

Two independent reviewers (W.L.Y and S.K) conducted data extraction. Data on the publication time, male to female incidence rate, follow-up time, data source (country, ethnicity), research institution, surgical approach, research type, and outcomes extracted from the eligible studies. The authors of the original articles were contacted if any discrepancies in data were present. If the opinions of two reviewers were inconsistent, the senior researcher (Z.J.M) was consulted.

### Validity Assessment

Two independent reviewers (W.L.Y and S.K) assessed the methodological quality of the articles using the Newcastle-Ottawa Scale (NOS). This scale determines the quality of non-randomized studies based on three categories (selection, exposure and comparability). It allocates zero or 1 point to each numbered item in each category with the exception of comparability where up to 2 points can be assigned. Each study can be allocated a maximum of 9 points, and a score of fewer than 7 points may signify a high risk of bias (10). The NOS was selected because it is validated and adaptable to our meta-analysis.

### Inclusion and Exclusion Criteria

#### Types of Studies

The criteria for eligible articles were studies describing outcomes of surgery for NTOS, published in English, human studies, with the full text available. The exclusion criteria were: (1) a review or meta-analysis, (2) consensus, treatment guidelines, (3) case-series and case reports (*N* < 10), (4) studies of endoscopic-assisted or robotic endoscopic-assisted transaxillary first rib resection (TFRR), (5) lacked a control group or were without postoperative follow-up evaluation.

#### Types of Participants

We included all studies with patients receiving operative interventions for TOS of any type. However, we excluded papers describing patients with compression due to malignancies in athletes and TOS in children (age <12 years). All participants had to be older than 12 years with poor results from conservative treatment or worsening symptoms more than 6 months before surgery. There was no restriction for sex, socioeconomic status, method of diagnosis, or duration of symptoms, but the follow-up period was set at more than 12 months. The lace was classified according to the definition given by the Oxford Center for Evidence-Based (11). Help from medical professionals who were fluent in both English and the language of the desired publication in times when the authors lacked fluency in the latter. Any inconsistencies within the included works were resolved by contacting the authors of the original studies. Whenever the information could not be obtained, all the reviewers joined the assessment until a consensus was reached.

#### Outcome Measures

The primary outcome measures were results of surgical treatment that were reported using validated questionnaires and outcomes according to Derkash's classification or similar. It is divided into excellent result (E): no pain, easy return to preoperative professional and leisure daily activities; good result; (G): intermittent pain well tolerated, possible return to preoperative professional and leisure daily activities; fair result (F) intermittent or permanent pain with bad tolerance, difficult return to preoperative professional and daily activities; poor result (P): symptoms not improved or aggravated. We extracted excellent and good as improved function, fair and poor as no improvement.

The secondary outcome was relieved in pain at least 12 months after the intervention preferably measured as change on a validated visual analog scale (VAS) or similar.

#### Definition

Long term outcome: the outcome about evaluation for NTOS after surgery (>12 months).

Short term outcome: the outcome about evaluation for NTOS after surgery (1 week to 3 months).

Derkash's classification: it is used to assess perceived disability in patients with arm, shoulder, and hand problems. These assessment schemes can well reflect the function of the upper limb and categorize primary outcomes as “excellent,” “good,” “fair,” and “poor”.

### Statistical Analysis

Statistical analysis was conducted by two reviewers (W.L.Y and S.K). We used Revman5.3 by Cochrane. A random-effects model was used for pain relieve with validated visual analog scale (VAS) and fix-effects model was used for improvement of upper limb with Derkash's classification. While probing for heterogeneity between the included studies, the Chi^2^ test and the Higgins I^2^ statistic were used. Cochran's Q *P*-value of <0.10 for the Chi^2^ test was chosen to show significant heterogeneity between the studies. Higgins *I*^2^ values between 0 and 40% were considered “might not be important,” 30%−60% as “might show moderate heterogeneity,” 50%−90% as “may show large heterogeneity,” and 75%−100% as “may represent notable heterogeneity” (12).

### Publication Bias

We checked for publication bias by building a funnel plot to visually check for asymmetry.

## Results

### Study Identification

The study identification terminated the plan, and 3,225 records were found. According to the exclusion and inclusion criteria (*n* = 763), articles (*n* = 433), review articles (*n* = 472) and articles published without a control group (*n* = 1,363) were excluded. A total of 10 studies were included in this meta-analysis.

### Characteristics of Included Studies

The characteristics of the included studies were presented in Table 1. One was a randomized controlled trial (13), nine were prospective controlled observational studies (14–22). To evaluate the long-term outcome of patients with NTOS, other types of TOS were excluded. Finally, 1,255 cases were included, including 622 in the SNBP group and 633 in the TFRR group. Four studies (13, 15, 17, 22) described pain relief with improvement of vas visual analog scale. Finally, 843 cases were included, including 440 in the SNBP group and 403 in the TFRR group. There was no difference in age, sex ratio, or the number of people in the two groups (*P* > 0.05).

**Table 1:** The characteristics of included studies.

**Study**	**Country**	**Type of study**	**Number of patients**	**Average-age (year)**	**Outcome**	**Follow-up (month)**
Aboul Hosn (14)	USA	Retros-pective	82	32.2	Upper limb function (Derkash's classification) and Complication: 1. In the SNBP group, 60 out of 63 had improved upper limb function. Twenty-one out of 32 in TFRR improved upper limb function. 2. There were 30 cases of complications after SNBP, 25 cases of pneumothorax, 3 cases of hemothorax, and 2 cases of vascular injury. Fifteen cases of postoperative complications of TFRR, 11 cases of pneumothorax, 2 cases of hemothorax, 2 cases of hemopneumothorax, 1 case of vascular injury	17.9
Altobelli (15)	USA	Retros-pective	334	40	Vas score (visual analog scale) and upper limb function (Quick-DASH score): 1. The satisfaction rate of TFRR dropped to 45% (254 cases) at 24 months and to 38% (118 cases) after 36 months. 2. The satisfaction rate of SNBP was 80% (80 cases) at 12 months, 69% at 24 months, and 59% (45 cases) at 36 months. The difference were statistically significant (*P* < 0.05). The recurrence rate is higher (54%) in TFRR	25
Balci (16)	Turkey	Retros-pective	47	37.9	The changes of ulnar nerve conduction velocities (UNCV); complications; mortality; upper limb function (Derkash's classification): The results showed that there was no significant difference between the TFRR and SNBP	55
Bhattacharya (17)	UK	Retros-pective	70	37	Pain relieve (visual analog scale) and upper limb function (Derkash's classification): The results showed that postoperative pain was relieved and upper limb function was improved in both TFRR and SNBP	43
Cikrit (18)	USA	Retros-pective	37	37.5	Blood loss; complications; upper limb function (Cervical Brachial Score Questionnaire): The results showed that SNBP had less blood loss (61 vs. 218 cc), fewer complications (1 vs. 21), and higher improvement in upper limb function (100 vs. 83%) than TFRR	36
Degeorges (19)	France	Retros-pective	176	35.7	Complications and improvement of upper limb function (Derkash's classification): The final follow-up was 69 cases in the SNBP group and 107 TFRR cases. The results showed that upper limb function improved in 43 cases in SNBP and 49 cases in TFRR	90
Nasim (20)	USA	Retros-pective	34	37	The upper limb function (Derkash's classification): Among the follow-up cases, 24 in SNBP and 13 in TFRR. The results showed that 20 cases in the SNBP improved, seven cases in the TFRR improved	>12
Parry (21)	England	Retros-pective	26	36.2	Upper limb function (Quick-DASH score): Among the cases, 13 in SNBP and 12 in TFRR. The results showed that upper limb function improved in 11 cases in SNBP and 8 cases in TFRR	>12
Sanders (22)	USA	Retros-pective	491	34	Pain relieve (visual analog scale) and upper limb function (Quick-DASH): Among the follow-up cases, there were 279 cases in the SNBP and 111 cases in the TFRR. After SNBP, 173 cases of pain were relieved and upper limb function were improved; 66 cases of TFRR achieved the same results, and there was no statistical difference in pain relief and improvement of upper limb function between the two	24
Sheth (13)	USA	Randomized -study	47	37	Vas score (visual analog scale): There was a statistically significant difference between TFRR and SNBP (*P* = 0.03)	47

Altobelli et al. (15) conducted a retrospective analysis of the postoperative effects of 254 CASES of NTOS. Vas visual analog Scale and Derkash's Classification were used to compare the improvement of upper limb function and pain relief after surgery, and the results showed that the satisfaction rate of TFRR was 53% at 12 months after surgery and decreased to 45% at 24 months and 38% at 36 months. The satisfaction rate was 80% at 12 months, 69% at 24 months, and 59% at 36 months after SNBP surgery. There were significant differences in upper limb function improvement and pain relief between the two methods (*P* < 0.05). In addition, the TFRR group had a higher postoperative recurrence rate (54%), requiring SNBP treatment again.

Aboul Hosn et al. (14) conducted a retrospective study on the postoperative follow-up results of 95 CASES of NTOS, including 63 cases of SNBP and 32 cases of TFRR. The follow-up time was over 24 months. Derkash's classification was used to evaluate the postoperative upper limb function, and the incidence of complications was compared between the two. The results showed that there were 30 complications after SNBP, including pneumothorax in 25 cases, hemothorax in three cases and vascular injury in two cases. Postoperative complications of TFRR included 15 cases, 11 cases pneumothorax, two cases hemothorax, two cases hemopneumothorax, and one case vascular injury. Postoperative upper limb function improved in 60 cases with SNBP and 21 cases with TFRR.

Bhattacharya et al. (17) retrospectively observed 60 patients after NTOS using the Derkash's Classification scale questionnaire, with a follow-up rate of 90% and a mean follow-up period of 43 months. The results showed that postoperative pain was relieved in TFRR and SNBP groups, and upper limb function was improved to some extent. There was no difference in long-term results between the two methods, and postoperative complications were not clearly described.

Sanders et al. (22) reviewed 491 NTOS patients with follow-up >24 months for postoperative evaluation. There were 279 cases in the SNBP group and 111 cases in the TFRR group. The upper limb pain was relieved and the function was improved in 173 cases after SNBP. The same results were obtained in 66 cases of TFRR, and there was no statistical difference in pain relief and upper limb function improvement between the two methods.

Sheth et al. (13) was the only RCT article in which the included patients were divided into two groups according to a completely randomized control, with 24 patients in the TFRR group and 25 patients in the SNBP group. The mean follow-up time was 37 months, and the postoperative efficacy was evaluated by VAS. Results Preoperative VAS scores were 77 ± 3 in TFRR group and 82 ± 3 in SNBP group, with no difference between the two groups (*P* = 0.28). Postoperative VAS scores of TFRR group (39 ± 7) and SNBP group (61 ± 7) were significantly different (*P* = 0.03).

### Test of Heterogeneity

We performed statistical testing for heterogeneity to decide if the long-term outcomes of surgical treatment were the same in the included studies. The Cochran Q result showed that there was mild heterogeneity between studies (χ^2^ = 11.90, *P* = 0.22). In addition, *I*^2^ revealed that 24% of the variation across the studies was because of heterogeneity rather than sampling error and chance.

### Quality Assessment

The mean Newcastle-Ottawa Scale score for 10 retrospective studies was 8.0, with higher scores showing better quality (Table 2).

**Table 2:** The Newcastle-Ottawa Scale score for studies.

**Qua Quality assessment of Newcastle-Ottawa Scale score ty Assessment of the Studies Using Newcastle-Ottawa Scale for Case-Control Studies**										
**Study number**	**First author**	**Case definition adequate**	**Representativeness of cases**	**Selection of controls**	**Definition of controls**	**Comparability**	**Ascertainment of exposure**	**Same ascertainment method**	**Nonresponse rate**	**Total**
1	Altobelli (15)	*	*	*	*	*	*	*	*	8
2	Aboul Hosn (14)	*	*	*		*	*	*	*	7
3	Balci (16)	*	*	*	*	**	*	*	*	9
4	Bhattacharya (17)	*	*	*		*		*	*	6
5	Cikrit (18)	*	*	*	*	**	*	*	*	9
6	Degeorges (19)	*	*	*	*	*	*	*	*	8
7	Nasim (20)	*	*	*	*	*	*	*	*	8
8	Parry (21)	*	*	*		*	*	*	*	7
9	Sanders (22)	*	*	*	*	*	*	*	*	8
10	Sheth (13)	*	*	*	*	**	*	*	*	9

*A maximum of one “star” for each item within the “Selection” and “Exposure/Outcome” categories; maximum of two “stars” for “Comparability.”*

### Improvement of Derkash's Classification

**Figure 2:**
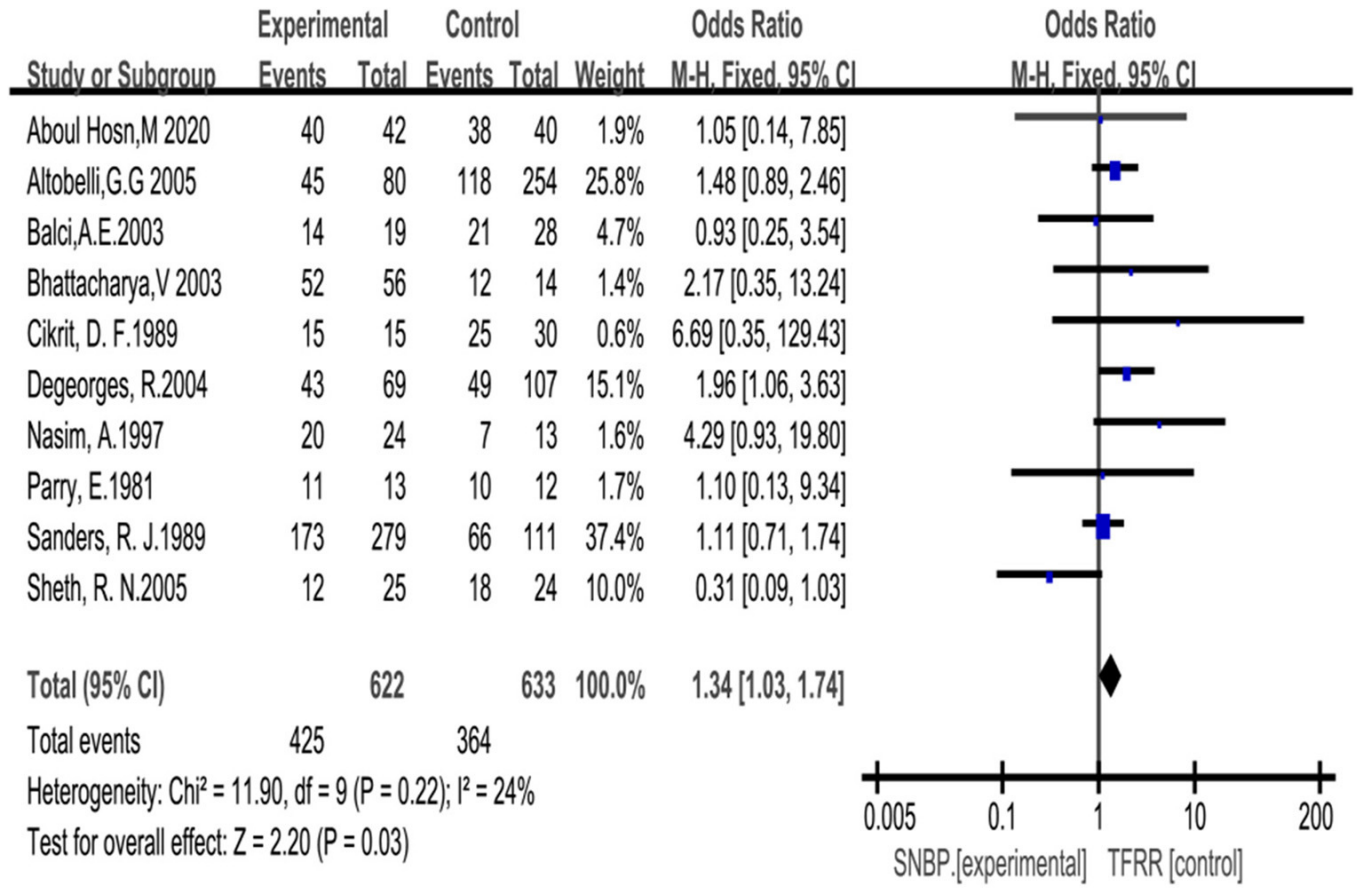
Forrest plot for improvement of upper limb function with SNBP and TFRR. CI, confidence interval.

Ten studies (*n* = 1,255 cases) were included, 622 in the SNBP group and 633 in the TFRR group. According to Derkash's classification, improvements in the upper limb were achieved in 425 cases with SNBP and 364 cases with TFRR. The excellent and good rate of SNBP was 1.34 times that of TFRR.OR = 1.34 (95% CI: 0.94, 1.92), *P* = 0.03 (Figure 2).

### Improvement of Vas Visual Analog Scale (VAS Score)

**Figure 3:**
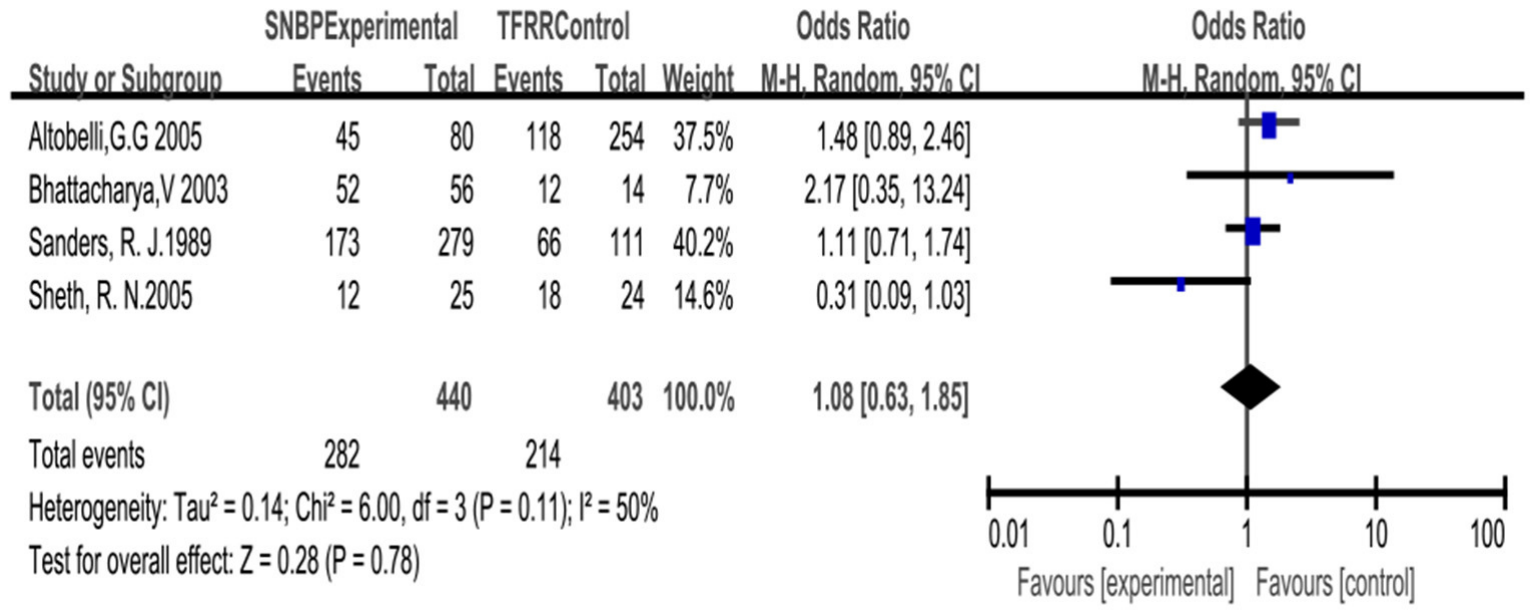
Forrest plot for Relief of pain with SNBP and TFRR. CI, confidence interval.

Based on the random-effects model for four studies with the changes of VAS. We note that 282 patients in the SNBP group had significant relief of pain and that there was no need to continue taking analgesics or reduce the dose for these patients; 214 patients in the TFRR group gained similar results. There was no difference between the two. OR = 1.08 (95% CI: 0.63, 1.85), *P* = 0.78 (Figure 3).

### Publication Bias

**Figure 4:**
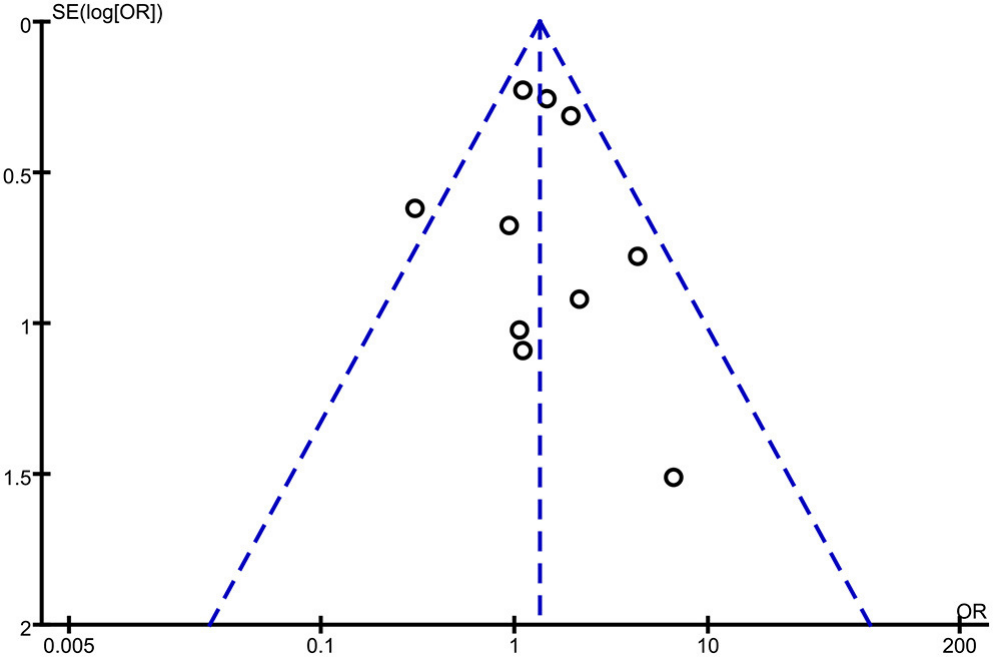
Funnel plot for improvement of upper limb function with surgery.

We set up a funnel plot to assess publication bias. In the non-publication bias, we would expect the studies to be symmetrical about the combined effect size. The remarked asymmetry in our funnel plot suggested the likelihood of some publication bias (Figure 4). This was mostly true when the analysis included clinical trials to find bias in publishing null results. However, regarding our research, this meant that including better-powered studies might have led to low-bias meta-analysis. The publication bias section and the idea of having a funnel plot was a technique to highlight any statistically probable error in the review, aiming to raise the truth of the conclusion drawn.

## Discussion

### Importance of Long-Term Outcome Assessment

Neurothoracic outlet syndrome has been perplexing clinicians for effective treatment because of the lack of Consistent and objective examinations (23). For patients who have been treated conservatively but ineffectually for more than 6 months, most clinicians prefer surgical treatment. Certainly, the long-term outcome of surgery directly affects the evaluation for treatment, as it is more instructive and convincing than the short-term outcome. Although many patients have good short-term results in their upper limb after surgery, over time, the results will gradually worsen or recur. Yoshizumi et al. (24) believed that long-term evaluation after treatment can be more stable and objective. However, choosing the better surgery has always been controversial (16, 25). This study aimed to systematically analyze the studies and provide the most comprehensive data thus far on the long-term results of surgery to determine the difference.

### Improvement of Derkash's Classification

Derkash's classification is a region-specific tool, as it assesses perceived disability in patients with arm, shoulder, and hand problems. These assessment schemes can well reflect the function of the upper limb and are used to categorize primary outcomes as “excellent” if there was no pain and a return to preoperative status, “good” if intermittent pain was well tolerated and there was the possibility of a return to the preoperative status, “fair” if intermittent or permanent pain was not well tolerated and a return to the preoperative status was difficult, or “poor” if symptoms had not improved or were aggravated (26). Peek et al. (27) confirmed that Derkash's classification was an effective tool to evaluate the improvement of upper limb function because of its wide use in practice and the availability of the data. Ruopsa et al. (28) conducted a survey lasting for 12 years that included 94 NTOS patients evaluated through Derkosh's classification, The results showed that most patients had significant improvement by SNBP. He believed that resection of the first rib was unnecessary. Johansen et al. (29) confirmed this through more cases (504 cases). We included 1,255 cases in the study. The excellent and good rate of SNBP was 1.34 times that of TFRR. OR = 1.34 (95% CI: 0.94, 1.92), *P* = 0.03.

### Improvement of Vas Visual Analog Scale (VAS Score)

The VAS score is widely used in pain assessment and is accurate, simple, and highly sensitive (30). Sheth et al. (13) divided patients into two groups according to a randomized control. The results showed that postoperative VAS scores in the TFRR (39 ± 7) and SNBP (61 ± 7) groups were significantly different (*P* = 0.03). However, this study had selection bias and few cases (45 cases), so it could not be concluded that TFRR was better than SNBP in relief pain. We included 843 cases in the study, the cases were enormous, there was no significant difference between the two.

### The Difference Between TFRR and SNBP

In the past, most doctors believed the occurrence of NTOS was related to compression of the brachial plexus by the first rib (31). Therefore many surgical approaches for removing the first rib, especially complete or partial removal of the first rib became popular (32, 33). TFRR achieved good results (48%), but the recurrence was still high (60%−70%) and often caused damage to the vessel and brachial plexus (34). Some studies showed that the NTOS was more likely to be related to the entrapment of the scalene muscles and bands on the brachial plexus. SNBP exposed the brachial plexus more fully, removed the scalene muscles and abnormal bands more thoroughly, and the effect was more precise than TFRR (35). However, scholars who agreed that the occurrence of NTOS stemmed from the first rib believed that TFRR could be more suited for skeletal compression (36).

Knowledge of the anatomy of the brachial plexus and thoracic outlet revealed that hypertrophy of the scalene muscle, fibrosis, and band compression were essential causes of neurovascular compression (37). Therefore, it was difficult to release the compression where the C5-T1 rami roots passed through the intervertebral foramen through TFRR, which was the main reason that the compression couldn't be relieved. SNBP can more fully disclose the C5-T1 roots by removing part of the scalene muscles, especially the portion where the brachial plexus penetrates the intervertebral foramen. The intraoperative release was more complete, and there was no need to remove the first rib, which avoided neurovascular damage and safer.

Our study does have several limitations. First, most studies were retrospective, only 1 randomized controlled trial was identified. the included studies are of moderate to poor methodological quality,as assessed using the NOS score;therefore, the overall quality of the available evidence is low, and there is a high risk of bias. Our analysis relied on these reported data, but we cannot improve the quality of the data. Second, the lack of unified evaluation criteria for NTOS creates a risk of bias. There are many evaluation criterias for improvement in upper limb, such as Derkash's Classification, Quick Shoulder and Hand questionnaire (Quick-dash Score) and Cervical Brachial Score Questionnaire (CBSQ). Derkash's classification was selected as the primary outcome measure in our analysis, other evaluation systems were excluded. Therefore, perhaps some interesting studies might have been ignored in this review. However, we listed all excluded studies with their characteristics and reason for exclusion. Third, this meta-analysis was limited because of the lack of objective evaluation criteria among the included studies. Although we conducted a comparison with ulnar nerve conduction velocities (UNCV), data on this topic are rare.

Finally, our results focus on patient reports of improvment in upper limb. Although VAS Score was used to analyze pain relief, only a few studies had objective data. Comparison using more objective evaluation criteria such as UNCV changes and VAS Score will make outcome more convincing. In addition, retrospective studies lack randomized controls, which has a high risk of bias. Multicenter, randomized controlled studies are needed in the future.

In conclusion, this meta-analysis shows that both SNBP and TFRR are effective for NTOS, but that SNBP is better than TFRR in improving Derkash's classification in the long term. Although patients treated with SNBP were more satisfactory, there was no significant difference in vas visual analog scale from TFRR.

## Author Contributions

WL is responsible for literature collection, data analysis, and writing articles. ZJ and WL was in charge of experimental design. ZJ and SK was responsible for evaluation of literature quality. SK was responsible for analyzing the data. QY and QJ are responsible for literature search and data collection. All authors contributed to the article and approved the submitted version.

## Funding

This work was funded by Science and Technology Research Program (1598011-5) and Science and Technology Department of Guangxi Zhuang Autonomous Region.

## Conflict of Interest

The authors declare that the research was conducted in the absence of any commercial or financial relationships that could be construed as a potential conflict of interest.

## Publisher's Note

All claims expressed in this article are solely those of the authors and do not necessarily represent those of their affiliated organizations, or those of the publisher, the editors and the reviewers. Any product that may be evaluated in this article, or claim that may be made by its manufacturer, is not guaranteed or endorsed by the publisher.
